# Prognostic Value of Erythrogram Indicators and C-Reactive Protein Levels in Predicting Outcomes of Patients with Coronavirus Disease 2019

**DOI:** 10.3390/ijms26094135

**Published:** 2025-04-27

**Authors:** Maria Eduarda Andretta, Matias Nunes Frizzo, Pauline Brendler Goettems-Fiorin, Thiago Gomes Heck, Lucas Machado Sulzbacher, Maicon Machado Sulzbacher, Mirna Stela Ludwig, Gaia Favero, Rita Rezzani, Vitor Antunes de Oliveira

**Affiliations:** 1 Department of Biomedicine, Regional University of Northwestern Rio Grande do Sul State (UNIJUÍ), Rua do Comércio, 3000-University, Ijuí 98700-000, RS, Brazil; maria.andretta@sou.unijui.edu.br; 2 Research Group in Physiology, Department of Life Sciences, Regional University of Northwestern Rio Grande do Sul State (UNIJUÍ), Rua do Comércio, 3000-University, Ijuí 98700-000, RS, Brazil; matias.frizzo@unijui.edu.br (M.N.F.); pauline.goettems@unijui.edu.br (P.B.G.-F.); thiago.heck@unijui.edu.br (T.G.H.); lucas.sulzbacher@unijui.edu.br (L.M.S.); maicon.sulzbacher@unijui.edu.br (M.M.S.); ludwig@unijui.edu.br (M.S.L.); 3 Postgraduate Program in Integral Attention to Health (PPGAIS-UNIJUÍ/UNICRUZ/URI), Rua do Comércio, 3000-University, Ijuí 98700-000, RS, Brazil; 4 Postgraduate Program in Pharmacology, Federal University of Santa Maria (UFSM), Av. Roraima, 1000-Camobi, Santa Maria 97105-900, RS, Brazil; 5 Anatomy and Physiopathology Division, Department of Clinical and Experimental Sciences, University of Brescia, 25123 Brescia, Italy; gaia.favero@unibs.it; 6 Interdipartimental University Center of Research “Adaption and Regeneration of Tissues and Organs (ARTO)”, University of Brescia, 25123 Brescia, Italy; 7 Italian Society of Orofacial Pain (SISDO), 25123 Brescia, Italy

**Keywords:** hematology, C-reactive protein, erythrogram indicators, red cell distribution

## Abstract

Coronavirus disease 2019 (COVID-19) has posed unprecedented challenges to global public health, highlighting the importance of prognostic biomarkers in critically ill patients. The oxidative stress developed in COVID-19 is associated with impairment in various human organs and systems, and it is related to erythrocyte injury, leading to an elevation in red cell distribution width (RDW) and systemic inflammation. This study aims to assess the prognostic value of erythrogram indicators and C-reactive protein (CRP) levels in 91 intensive care unit-admitted COVID-19 patients, categorized into survivor patients (discharge group) and non-survivor patients (death group). The results were presented using descriptive statistics and the Mann–Whitney test. The most severe cases of respiratory failure in which the patients did not survive showed higher red cell distribution width (RDW) and lower values of red blood cell count, hemoglobin, and hematocrit. RDW may be an important indicator of mortality, as demonstrated by the receiver operating characteristic (ROC) curve analysis. Furthermore, this increase in RDW is correlated with elevated CRP levels, another important clinical outcome for these patients. In conclusion, elevated RDW and CRP levels at admission may be reliable predictors of unfavorable outcomes, emphasizing the utility of these indicators in clinical assessments of COVID-19 patients.

## 1. Introduction

Despite the decrease in acute Coronavirus disease 2019 (COVID-19) cases, the impact of SARS-CoV-2 pandemic also remains significant in the fields of respiratory medicine and the management of infectious diseases. The identification of reliable biomarkers is essential for understanding severe respiratory conditions, particularly in the context of acute respiratory syndrome caused by viral infections. The present study examines the prognostic value of erythrogram indicators and C-reactive protein (CRP) levels in intensive care unit (ICU) patients affected by COVID-19, emphasizing their role in predicting severe clinical outcomes. These indicators are often associated with the processes of inflammation and oxidative stress [1,2].

In the pathophysiology of COVID-19, it is known that the viral infection triggers an intense inflammatory process. Systemic inflammation has been shown to be associated with poor outcomes in patients with COVID-19 [3]. This excessive inflammatory response can result in pulmonary edema, fibrosis, and thrombosis, resulting in hypoxia, acute respiratory distress syndrome, multi-organ failure, and even death [4]. Furthermore, alleviation of the systemic inflammatory response could be fundamental for COVID-19 patients, and some controlled trials have shown the value of different anti-inflammatory agents [5,6]. The exacerbated pro-inflammatory reaction, known as the “cytokine storm”, developed by leukocytes, upon recognizing the viral antigen, alters several hematological parameters, such as neutrophil, lymphocyte, and platelet counts. This allows for the monitoring of inflammatory markers, such as the neutrophil-to-lymphocyte ratio (NLR), platelet-to-lymphocyte ratio (PLR), and systemic immune-inflammation index (SII—calculated as platelets multiplied by neutrophils and divided by lymphocytes). However, these markers have not yet shown a consistent association with the risk of in-hospital mortality, unlike CRP, which can be used as a mortality biomarker in patients with greater frailty and worse prognosis in COVID-19 [7]. Several different inflammatory biomarkers (such as cytokines and complement proteins) were studied to address the relationship between inflammation and COVID-19 severity, and they aim to improve prognostication and to development specific therapies [8,9]. Recently, Bastos Mendes et al. (2025) [8] applied promising machine learning algorithms to generate models capable of predicting mortality in COVID-19 patients, also providing deeper insights into the complexity of cytokine interactions when comparing survivor and non-survivor COVID-19 patients. Previous research has highlighted the importance of hematological markers, such as red cell distribution width (RDW) and hemoglobin (HBG), in predicting mortality across various medical conditions [9,10]. In patients with severe respiratory diseases, fluctuations in these markers may indicate progression to critical states, including acute respiratory distress syndrome and multiple organ failure [11,12]. Oxidative damage may influence these markers, reflecting the severity of the clinical condition and potentially serving as a prognostic indicator of adverse outcomes [13,14,15]. The increased production of reactive oxygen species (ROS), in relation to a deficiency in antioxidant defenses, leads to oxidative stress, which is associated with the development of erythrocyte damage and an elevation in RDW, a marker that reflects variation in RBC size [16].

Thus, this study aims to assess whether significant alterations in erythrogram indicators and CRP levels in ICU patients with acute respiratory syndrome related to COVID-19 can serve as effective prognostic biomarkers for severe outcomes. By doing so, we hope to provide critical insights for monitoring and managing intensive care patients, thereby enriching the medical literature with valuable information that may enhance therapeutic strategies in challenging health contexts.

## 2. Results

### 2.1. Characteristics and Outcome of Study Participants

Among the 91 patients included in the study, 36 were women with a mean age of 55.2 (±16.37) years, and 55 were men with a mean age of 53.2 (±14.51) years. A total of 81.3% of the patients were discharged, while 19.7% died. For the following analysis and comparisons, the patients were divided into two groups based on the final outcome of the disease—the survival group (discharge group) and the non-survival group (death group).

### 2.2. Erythrogram Indicators in the Evaluation of COVID-19

**Figure 1:**
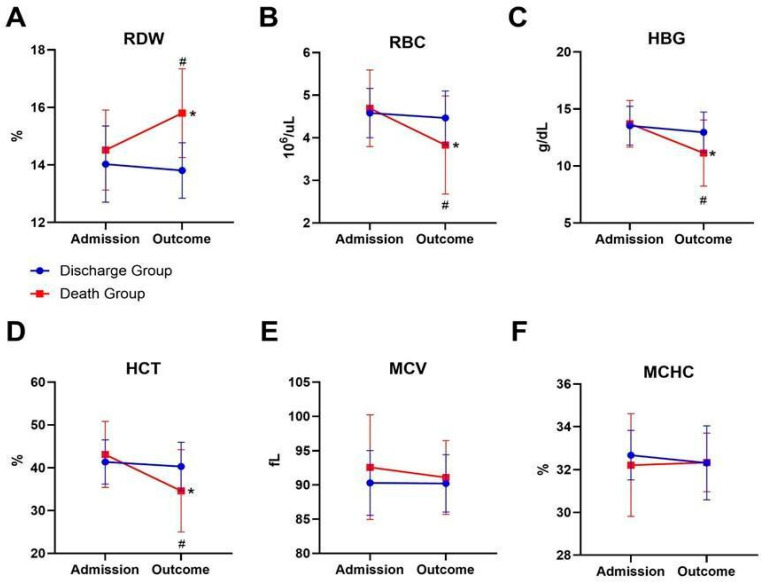
Comparison of erythrogram indicators between discharge group and death group immediately after admission. (**A**) Red cell distribution width (RDW), * *p* = 0.0134 (admission: death group vs. outcome: death group), and # *p* < 0.0001 (outcome: discharge group vs. outcome: death group). (**B**) Red blood cells (RBCs) count, * *p* = 0.0025 (admission: death group vs. outcome: death group), and # *p* = 0.0053 (outcome: discharge group vs. outcome: death group). (**C**) Hemoglobin (HGB), * *p* = 0.007 (admission: death group vs. outcome: death group), and # *p* = 0.0025 (outcome: discharge group vs. outcome: death group). (**D**) Hematocrit (HCT), * *p* = 0.005 (admission: death group vs. outcome: death group), and # *p* = 0.0038 (outcome: discharge group vs. outcome: death group). (**E**) Mean corpuscular volume (MCV), *p* = 0.456. (**F**) Mean corpuscular hemoglobin concentration (MCHC), *p* = 0.418. Discharge group *n* = 74 and death group *n* = 17. The statistical analysis was performed using a two-way ANOVA with Tukey’s post hoc test.

The discharge group presented an increase in RDW when comparing admission values with those at the time of outcome (Figure 1A), along with a decrease in red blood cell (RBC) count (Figure 1B), HBG (Figure 1C), and hematocrit (HCT) (Figure 1D). Thus, at the time of outcome, patients who did not survive had higher RDW (Figure 1A) and lower RBC count, HBG, and HCT values (Figure 1B–D) compared to patients who were discharged. However, mean corpuscular volume (MCV) (Figure 1E) and mean corpuscular hemoglobin concentration (MCHC) (Figure 1F) did not exhibit significant changes among the discharge group and the death group.

### 2.3. Red Cell Distribution Width and C-Reactive Protein as COVID-19 Indicators of Severity

**Figure 2:**
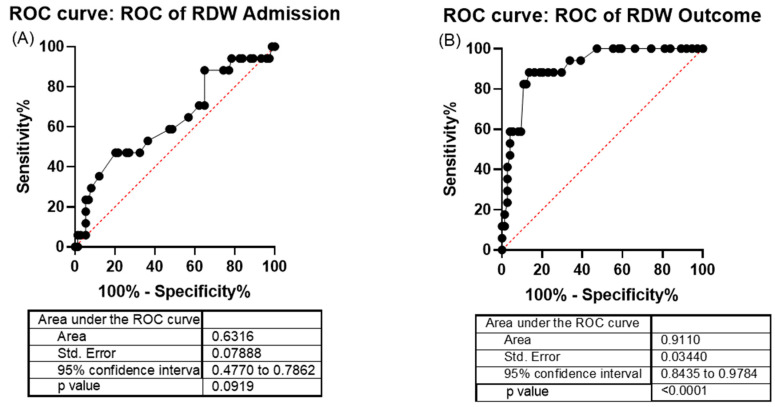
Receiver operating characteristic (ROC) curve of red cell distribution width (RDW) parameters of discharge group and death group. (**A**) Receiver operating characteristic (ROC) curve of red cell distribution width (RDW) at patient’s admission. (**B**) Receiver operating characteristic (ROC) curve of red cell distribution width (RDW) at patient’s outcome. Discharge group *n* = 74 and death group *n* = 17.

RDW was not a risk indicator at patient admission (Figure 2A). However, RDW can be considered a predictor of severity in COVID-19. In fact, when evaluating a patient’s outcome (discharge group or death groups), RDW can serve as an indicator of mortality, as evidenced by the receiver operating characteristic (ROC) curve test (*p* < 0.0001), with an area under the curve (AUC) of 0.91 (reflecting a 90% chance of correct classification), using a cutoff value above 14.75 (sensitivity of 88% and specificity of 86%) (Figure 2B).

**Figure 3:**
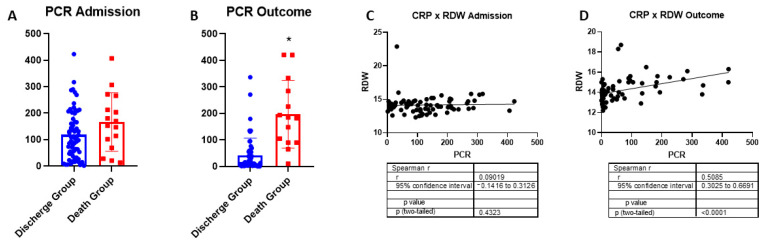
Comparison of C-reactive protein (CRP) levels between discharge group and death group. (**A**) CRP levels after hospital admission and Mann–Whitney test *p* = 0.096. Discharge group *n* = 63 and death group *n* = 16. (**B**) CRP levels before the outcome, Mann–Whitney test * *p* < 0.0001. Discharge group *n* = 55 and death group *n* = 14. (**C**) Correlation between CRP levels and RDW at patient’s admission, Spearman’s rank correlation test r = 0.090, and *p* = 0.4323. (**D**) Correlation between CRP levels and RDW at patient’s outcome, Spearman’s rank correlation test, r = 0.508, and *p* < 0.0001.

No changes in circulating CRP were observed at admission, while it increased in the patients in the death group (Figure 3A,B, respectively). In turn, this inflammatory response marker did not correlate with RDW at admission. However, the increase in systemic inflammation caused by COVID-19 was correlated with greater variability in RDW at the time of outcome (Figure 3C,D, respectively).

## 3. Discussion

RDW increased significantly in the non-survival group, indicating anisocytosis, a marker of anemia, in our patients. Anisocytosis is usually associated with increased all-cause mortality and abnormalities in erythrocyte size distribution have been associated with inflammation [17]. The RDW is a well-known nonspecific marker of general illness, and the specific mechanisms for the RDW alteration associated with COVID-19 remain not fully elucidated, even in studies that clearly show evidence of this phenomenon in COVID-19 [18]. However, some particularities may help to understand the scenario, as the specific binding of the SARS-CoV-2 to band-3 RBC proteins distinguished from other cells, the RBCs have no ACE2; thus, the binding of the virus and RBCs specifically may cause protein oxidation and the fragmentation of integral (e.g., glycophorin and band 3) and peripheral (e.g., spectrin) proteins, impairing ion exchange and the flexibility of these cells, despite normal RBC and hematocrit levels [19,20]. Zhang et al. (2020) [21] performed a comprehensive meta-analysis in which the prognostic role of RDW in patients with sepsis was evaluated. Interestingly, the authors reported that higher levels of RDW at the admission time point can be considered a powerful predictor of mortality in sepsis, a disease that shares several inflammatory characteristics as COVID-19. The RDW value may reflex the presence of inflammatory response, which can negatively affect the bone marrow function, iron metabolism, and red blood cell homeostasis [22], then leading to blunted erythropoiesis (anisocytosis). Furthermore, oxidative stress reduces RBC survival and increases the release of large premature RBCs into the peripheral circulation, which directly leads to elevated RDW [21,23]. An elevated RDW resulted in a 2- or 6-fold increase in the relative mortality of COVID-19 patients, dependent on basal RDW levels, with higher basal levels representing the worst scenario. RDW alterations can be associated with other biomarkers or confounding factors, but including this variable in prognostic models may have benefits in term of prognostic value [24].

Recently, Hakobyan et al. (2025) [25] observed in 74 COVID-19 patients that CRP and anisocytosis levels are closely related to the severity of SARS-CoV-2 infection and that elevated ferritin levels were associated with anisocytosis, altered HBG microspectrophotometric characteristics, and disease adverse outcomes. Despite a strong correlation between elevated anisocytosis and CRP, this phenomenon is only part of the complicated erythrocyte pathology resulting from SARS-CoV-2 infection.

It is estimated that the increase in RDW is related to underlying acute or chronic diseases [15,26]. According to previous studies, in the context of COVID-19, the increase in RDW showed a statistically significant association with mortality and can be used as a predictor of prognosis in critically ill patients [26,27,28]. The underlying mechanisms for this alteration are not well defined, but an increase in reticulocytes may be one of the contributing factors [10,13].

According to Wang et al. (2020) [27] and Linssen et al. (2020) [29], changes in erythrogram indicators are heterogeneous and related to the severity of COVID-19, comorbidities, and patient age. The main abnormality is the decrease in HGB levels, which may indicate the development of anemia. In our study, the RBC count in patients who did not survive was lower compared to those who survived (discharge group). One of the factors contributing to this difference may be related to what Thomas et al. (2020) [30] previously suggested; SARS-CoV-2 infection causes significant damage to the homeostasis of RBCs membrane structure, caused by oxidative, glycolytic, and lipid imbalances. The viremia of SARS-CoV-2 can induce oxidative stress at the circulatory level. The virus attacks HGB groups in erythrocytes, so leading to the release of ferric ions from heme groups into the bloodstream, which causes oxidative damage to erythrocytes [31].

A previous study highlighted oxidative stress as an etiological factor for lipid peroxidation of the erythrocyte cell membrane, which is the primary cause of increased RDW [16]. This finding supports our results; the death group, at the time of outcome, presented a higher RDW with respect to the discharge group.

The decrease in RBC count is directly related to the decrease in HCT, as it represents the percentage of these cells in the total blood volume. This erythogram indicator impairment is consistent with a study conducted in Brazil, with 3014 individuals [31], in which it was reported that 9.5% of patients require an RBC transfusion. Furthermore, it can be observed that the findings of Dalmazzo et al. (2021) [32] and the data related to the present study are directly associated with the severity and mortality caused by COVID-19.

The HGB values in the death group were significantly lower compared to the discharge group, with a mean value of 11.24 g/dL and 12.88 g/dL, respectively, which already fall within the anemic range [33]. The reduction in this parameter is frequently found, and it is usually associated with severe illnesses. Furthermore, its progressive decrease can be a warning sign of negative clinical progression, and it is also associated with longer hospitalizations, poor clinical conditions, and high mortality rates [14,34,35,36,37]. The decrease in RBC count and HGB may be directly related to the COVID-19 inflammatory process, due to the action of immune factors, mainly interferons and interleukins, which inhibit the production of erythropoietin (EPO). EPO is a hormone produced by the kidneys responsible for the proliferation and differentiation of myeloid progenitor cells, which give rise to RBCs. Therefore, in the presence of increased inflammatory factors, the decrease in erythropoiesis due to reduced EPO promotes anemia [38,39,40]. Anemia is associated with a 2.6-fold increase in the risk of mortality in chronic obstructive pulmonary diseases. Due to the role of HGB in the transport of oxygen (O_2_) and carbon dioxide (CO_2_), a decrease in its concentration indicates that patients with anemia, whether caused by infection or not, may have a reduced capacity of HGB to meet the increased O_2_ demand in peripheral tissues, which occurs due to the hypermetabolic state caused by infection. Therefore, it is an important marker that should be monitored during hospitalization [9,41,42].

For the analysis of RDW performance, the ROC curve was used, which provides a graphical representation of a quantitative data model’s performance, according to its sensitivity and specificity rates. The AUC estimates the probability of correct classification of a subject by chance (test accuracy), and the AUC values are interpreted as follows: 0.5–0.6 (poor), 0.6–0.7 (fair), 0.7–0.8 (poor to good), 0.8–0.9 (good), and >0.9 (excellent) [43].

Monitoring RDW is an important erythrocyte indicator for individuals [44]. RBCs are responsible for oxygen transport in the blood, and this hematological parameter can indicate the risk for diseases, such as COVID-19, which is known to cause dysfunction in respiratory tissue [41]. Furthermore, a previous study showed that COVID-19 caused hematological dysfunction in adult cancer patients, demonstrating that an increase in RDW to a value of 16.9 has a sensitivity of 72.7% and specificity of 90% for severe cases of the disease, with an AUC of 0.841 (*p* = 0.008) [45]. An elevated level of RDW is considered a marker of severity in coronary artery disease with myocardial ischemia [46], and it is also associated with chronic respiratory diseases [47], which are comorbidities and risk factors for the development of more severe forms of COVID-19 [48]. Moreover, inflammatory processes are also marked by an increase in CRP level, which correlates with RDW elevation [49], as confirmed in the present study. The elevation of CRP in inflammatory processes is well-documented and suggests a systemic inflammatory state. In the present study, the significant correlation between CRP and RDW levels, especially before the outcome, reinforces the hypothesis that severe inflammation is related to hematological changes, so indicating a potentially worse prognosis in COVID-19 patients. This observation is consistent with previous research studies showing that CRP is not only an inflammatory marker but may also reflect the severity of the inflammatory response, which can be associated with oxidative stress and endothelial dysfunction [50,51].

Oxidative damage plays a critical role in the pathophysiology of acute respiratory diseases, including COVID-19. Chronic inflammation leads to the excessive production of ROS, which can damage cells and tissues. Studies have shown that CRP mediates the inflammatory response, and elevated CRP levels may indicate a higher production of ROS, reflecting an oxidative stress state [52]. This connection is important, as it suggests that increased CRP may be indicative not only of inflammation but also of significant oxidative injury. Therefore, the combination of high levels of CRP and RDW may indicate an exacerbated inflammatory response, where oxidative stress plays a fundamental role in the deterioration of the patient’s clinical condition [17,53,54].

**Figure 4:**
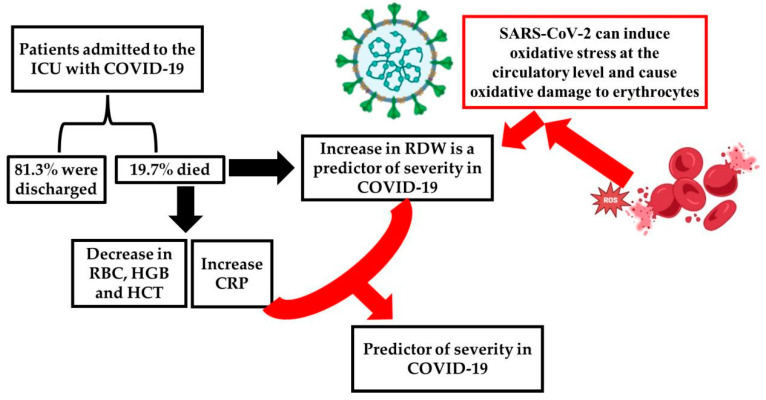
Summary of key evidence and study discussions. More severe clinical conditions with worse outcomes (death group) have higher RDW and CRP and lower RBC count, HBG, and HCT than patients who survive (discharge group). SARS-CoV-2 can induce oxidative stress at the circulatory level and cause oxidative damage to erythrocytes [17], which increases RDW. CRP is involved in mediating the inflammatory response, and elevated CRP levels may indicate increased ROS production, reflecting a state of oxidative stress, with the association of CRP and RDW potentially serving as predictors of COVID-19 severity [17,31]. Coronavirus disease 2019 (COVID-19); C-reactive protein (CRP); hematocrit (HCT); hemoglobin (HGB); intensive care unit (ICU); red blood cell (RBC); red cell distribution width (RDW). The illustration was generated using the BioRender virtual platform (https://www.biorender.com/ accessed on 24 April 2025).

The main evidence and discussions addressed in our study are illustrated in Figure 4.

Monitoring inflammatory variables, such as CRP, offers a level of sensitivity that allows for the accurate assessment of acute inflammatory diseases like COVID-19. However, certain factors—such as comorbidities, age, and microbial co-infections—can influence the inflammatory response, and consequently, evaluating some inflammatory markers, such as cytokines and CRP [55], will be an important point not thoroughly addressed in our study. The present study holds significant potential in contributing to the prognostic evaluation of patients affected by COVID-19 by analyzing data from a community-based laboratory during a critical phase of the pandemic marked by high mortality and using low-cost biomarkers. However, the present study has some limitations, particularly due to the lack of access to information on patient comorbidities and the absence of an analysis exploring the relationship between these biomarkers and patients’ clinical histories. Despite these limitations, RDW proved to be a relevant parameter for the laboratory monitoring of patients. This indicator, alongside other variables, is influenced by pathophysiological mechanisms associated with COVID-19, such as inflammation and oxidative stress, as previously discussed. The integrated use of these data may provide a more comprehensive approach to laboratory follow-up, complementing the clinical evaluation of patients.

These findings are consistent with previous studies that have identified RDW as a marker of severity in cases of respiratory failure, regardless of the presence of comorbidities, such as systemic arterial hypertension and diabetes, as well as variations in age and body mass index among patients [15].

Considering this, even in a community-based laboratory, RDW was shown to be relevant to monitoring patients. Together with other variables, RDW is affected by mechanisms related to the pathogenesis of COVID-19, such as oxidative stress, as previously discussed. This combined approach can contribute to more comprehensive laboratory monitoring with the clinical evaluation of patients [52]. This is consistent with a previous study in which RDW was a marker of severity in respiratory failure, even when the study population had different comorbidities (such as systemic arterial hypertension and diabetes) and variability in age and body mass index [15].

## 4. Materials and Methods

### 4.1. Experimental Design

**Figure 5:**
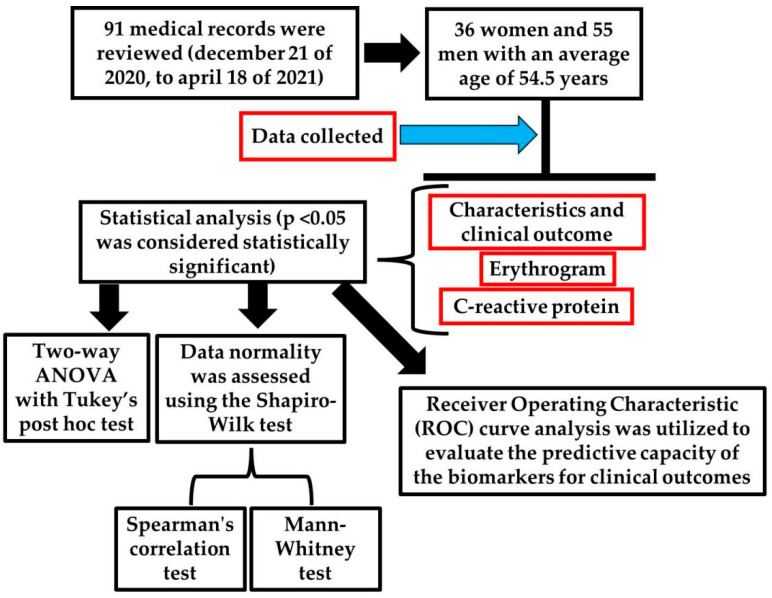
Study methodology. The illustration was generated using the BioRender virtual platform (https://www.biorender.com/ accessed on 24 April 2025). The red boxes indicated the bioindicators collected in the present study.

This study is part of an observational, descriptive, and analytical research conducted based on data collected from medical records of patients with confirmed COVID-19 diagnosis admitted to the ICU at Hospital Bom Pastor, located in the northwest region of the state of Rio Grande do Sul, Brazil. The inclusion period covers the entire duration from 21 December 2020 to 18 April 2021, corresponding to the time when the data collection were approved by the ethics committee (Figure 5). Data collection occurred in November 2021, subsequent to receiving approval from the Research Ethics Committee of UNIJUÍ, under approval number 5.073.813, in accordance with the ethical principles outlined by Resolution 466/2012 of the National Health Council. The requirement for informed consent was waived, as the study involved only the retrospective review of medical records without direct patient contact. Brazilian national legislation considers the approval of the Research Ethics Committee (CEP) and the Institution as appropriate measures to ensure compliance with ethical research principles for studies involving exclusively the collection of secondary data from medical records, without direct patient identification and without any intervention or interaction with them. This procedure aligns with Resolution 466/2012 of the National Health Council, which regulates research involving human subjects in the country.

A total of 91 medical records were reviewed, including 36 women and 55 men with an average age of 54.5 years. These records represent all patients admitted to the ICU during the study period who underwent a complete blood count on the day of admission and at the last examination before discharge from the ICU, regardless of the duration of their stay or whether discharged alive or deceased.

### 4.2. Patients Inclusion and Exclusion Criteria

The inclusion criteria specified patients with a COVID-19 diagnosis who were admitted to the ICU. Exclusion criteria included patients receiving outpatient care and those who did not undergo a complete blood count. The study focused on variables such as age, clinical outcomes, CRP, and erythrogram indicators on the day of admission and before discharge or death, to facilitate a comparative analysis.

### 4.3. Statistical Analysis

Statistical analyses were performed using Prism GraphPad 8.0 software. Hematimetric indices of the erythrogram were evaluated between groups and at both the time of admission and clinical outcome using a two-way ANOVA with Tukey’s post hoc test. To compare CRP values at admission and outcome, data normality was assessed using the Shapiro–Wilk test, and the Mann–Whitney test was used for data that did not follow a normal distribution. Spearman’s rank correlation was applied to assess the relationships between non-parametric continuous variables (CRP and RDW). ROC curve analysis was used to evaluate the predictive capacity of biomarkers for clinical outcomes. All tests were two-tailed, and a *p*-value of <0.05 was considered statistically significant.

## 5. Conclusions

It was found that shortly after hospitalization, patients who did not survive already exhibited higher levels of RDW compared to those who survived. Additionally, within the death group, these differences became more pronounced in the latest blood tests, showing a decrease in RBC count, HGB, and HCT, alongside an increase in RDW. Furthermore, the increase in CRP correlated with the rise in RDW, highlighting that the inflammatory state contributes to anisocytosis.

These findings suggest a significant interplay between inflammation and oxidative stress, indicating that elevated CRP levels may reflect not only systemic inflammation but also oxidative damage, which can exacerbate clinical deterioration. Therefore, the combination of increased RDW and CRP levels can serve as a valuable predictor of unfavorable prognosis in severely ill patients. This underscores the importance of monitoring these biomarkers to facilitate early interventions and improve clinical outcomes in critically ill individuals.

## Data Availability

The original contributions presented in the study are included in the article, and further inquiries can be directed to the corresponding author.
